# Organizational-Level Strategies With or Without an Activity Tracker to Reduce Office Workers’ Sitting Time: Rationale and Study Design of a Pilot Cluster-Randomized Trial

**DOI:** 10.2196/resprot.5438

**Published:** 2016-05-25

**Authors:** Charlotte L Brakenridge, Brianna S Fjeldsoe, Duncan C Young, Elisabeth A H Winkler, David W Dunstan, Leon M Straker, Christian J Brakenridge, Genevieve N Healy

**Affiliations:** ^1^ The University of Queensland School of Public Health Brisbane Australia; ^2^ Lendlease Sydney Australia; ^3^ Baker IDI Heart and Diabetes Institute Melbourne Australia; ^4^ School of Public Health and Preventive Medicine Monash University Melbourne Australia; ^5^ School of Exercise and Nutrition Sciences Deakin University Melbourne Australia; ^6^ Department of Medicine Monash University Melbourne Australia; ^7^ School of Sport Science, Exercise and Health The University of Western Australia Perth Australia; ^8^ Mary MacKillop Institute for Health Research Australian Catholic University Melbourne Australia; ^9^ School of Physiotherapy and Exercise Science Curtin University Perth Australia

**Keywords:** wearable device, self-monitoring, sedentary lifestyle, office workers, light intensity activity, ecological model, workplace, trial, objective, activity monitor

## Abstract

**Background:**

The office workplace is a key setting in which to address excessive sitting time and inadequate physical activity. One major influence on workplace sitting is the organizational environment. However, the impact of organizational-level strategies on individual level activity change is unknown. Further, the emergence of sophisticated, consumer-targeted wearable activity trackers that facilitate real-time self-monitoring of activity, may be a useful adjunct to support organizational-level strategies, but to date have received little evaluation in this workplace setting.

**Objective:**

The aim of this study is to evaluate the feasibility, acceptability, and effectiveness of organizational-level strategies with or without an activity tracker on sitting, standing, and stepping in office workers in the short (3 months, primary aim) and long-term (12 months, secondary aim).

**Methods:**

This study is a pilot, cluster-randomized trial (with work teams as the unit of clustering) of two interventions in office workers: organizational-level support strategies (eg, visible management support, emails) or organizational-level strategies plus the use of a waist-worn activity tracker (the LUMOback) that enables self-monitoring of sitting, standing, and stepping time and enables users to set sitting and posture alerts. The key intervention message is to ‘Stand Up, Sit Less, and Move More.’ Intervention elements will be implemented from within the organization by the Head of Workplace Wellbeing. Participants will be recruited via email and enrolled face-to-face. Assessments will occur at baseline, 3, and 12 months. Time spent sitting, sitting in prolonged (≥30 minute) bouts, standing, and stepping during work hours and across the day will be measured with activPAL3 activity monitors (7 days, 24 hours/day protocol), with total sitting time and sitting time during work hours the primary outcomes. Web-based questionnaires, LUMOback recorded data, telephone interviews, and focus groups will measure the feasibility and acceptability of both interventions and potential predictors of behavior change.

**Results:**

Baseline and follow-up data collection has finished. Results are expected in 2016.

**Conclusions:**

This pilot, cluster-randomized trial will evaluate the feasibility, acceptability, and effectiveness of two interventions targeting reductions in sitting and increases in standing and stepping in office workers. Few studies have evaluated these intervention strategies and this study has the potential to contribute both short and long-term findings.

## Introduction

### Background

The workplace is a key health promotion setting [1,2], with now extensive evidence demonstrating the effectiveness of interventions targeting physical activity in this environment [3]. Generally, physical activity programs in workplaces have had a beneficial impact on health risk biomarkers, work attendance, and job stress [3]. However, a common criticism has been that workplace physical activity interventions typically only reach those who are already fit and motivated to be active [4], and have had negligible impact on reductions in sitting [5,6]. These two issues may be addressed through a shift in focus to organization-wide interventions that have the potential to reach a greater proportion of employees than conventional individually centered approaches, and to interventions that target change across the activity spectrum including: sedentary time (sitting or lying with low energy expenditure, ≤1.5 metabolic equivalents (METS) [7]); light intensity activities (such as standing or incidental movement, >1.5 to <3 METS [8]); as well as moderate- and vigorous-intensity physical activities (MVPA, ≥3 to <9 METS [8]).

Sedentary and light intensity behaviors occupy much of the waking day [8], more than 95% on average in adults. The distribution of time spent between these two behaviors is increasingly being recognized as having potentially important implications for health and well-being [9,10]. Higher levels of light intensity activity are associated with improved blood glucose levels [11], physical health and well-being [12], and decreased depression [13]. Although several behaviors fall within the light intensity spectrum, there is preliminary evidence to suggest that even just postural shifts to standing could have some metabolic benefit [14,15]. In contrast, high levels of daily sitting time have been detrimentally associated with outcomes such as all-cause mortality, cardiovascular disease incidence and mortality, type 2 diabetes incidence [16,17], and cancer incidence and mortality [17], as well as risk indicators for these [18]. In addition, there is growing evidence suggesting that the manner in which sitting is accumulated may be important, with more breaks (or interruptions: either with activity or standing) in sedentary time showing beneficial associations with cardiometabolic indicators including waist circumference [15,19,20], blood glucose [15,19-22], insulin [21,22], and triglycerides [15,18-20].

### Sitting in the Workplace

For office workers, workplace sedentary time is a large contributor to overall sedentary time [23,24], with studies showing that office workers spend, on average, three-quarters of their work hours sitting [23-27]. Given this pervasive nature of sitting in the office, and that office workers are the largest individual occupational sector [28], the office workplace has been identified as a key setting to target reductions in sitting time [29].

Office workers generally have the advantage of being colocated, which means a wide range of influences can be targeted within intervention approaches. Based on workplace health promotion frameworks [1,30] and behavioral models for understanding sitting and activity [31,32], common influences are organizational, environmental, and individual factors, with the notion that individual-level strategies alone are unlikely to be sufficient for sustained behavior change [32]. Organizational-level strategies are seen as particularly important for program implementation [33], to change the culture of an organization [30,34], and for programs to be institutionalized into the organization and sustained [30]. Key organizational-level strategies include having management support for programs [33,34] and implementing the program from within the organization via dedicated onsite staff or workplace ‘champions’ [35].

Despite these frameworks, workplace interventions targeting MVPA have typically targeted the individual and not the organizational- or environmental-level influences [36,37]. Similarly, there have been very few studies that have implemented organizational-level strategies in workplace sitting interventions. The studies that have addressed the organizational level have done so in combination with individual-level strategies (eg, health coaching) and/or physical environment (eg, sit-stand workstation) strategies [27,38-41]. Findings from these multicomponent interventions have shown significant and large reductions in sitting both at the workplace (eg, −125 minutes/8-hour workday [38], −89 minutes/8-hour workday [39]) and across the whole day (eg, −66 minutes/day [40]). While organizational elements were reported as important in these studies [38,39], and a lack of management support reported as a key issue for those studies that have reported less success [27,41], no study to date has identified how much change results from the organizational-level component alone. If found to be effective, an added benefit of this organizational-level approach is that the intervention elements are minimal both in terms of cost and employee burden, which may be beneficial for organizations that have limited employee time or funds.

### Activity Trackers as Intervention Tools

While organizational-level strategies may be sufficient on their own, it is also important to consider individualized elements to possibly enhance the success of the intervention. Evidence suggests that individual, self-monitoring devices such as pedometers are a common element of successful workplace physical activity interventions [36,42] that can increase activity [43-48] and decrease sedentary time [43,44]. Recent advances in technology have seen the emergence of more sophisticated wearable activity trackers that go beyond just simple step counting to incorporate many of the strategies known to support behavior change [49]. Such strategies, including the provision of detailed, real-time feedback, long-term tracking, prompts, and goal setting, as well as the measurement of multiple behaviors, give activity trackers the potential to be effective behavior intervention tools [49,50]. Indeed, their potential as low-cost behavior change support tools has been recognized by several workplace wellness programs in the United States, where activity trackers are distributed to encourage employees to get healthy and reduce their insurance premiums [51,52].

There has been minimal research on the feasibility, acceptability, and effectiveness of activity trackers as intervention tools. A recent review highlighted the large heterogeneity in the small field of research studies and the mixed quality of the research [53]. However, there was some indication that activity trackers may lead to pre-post physical activity increases and are feasible to wear [53]. There was, however, very limited evidence for long-term physical activity increases [53]. In fact, only four studies out of the 11 in the review evaluated long-term outcomes (≥6 months), with only one study resulting in a significant change in physical activity [53]. There is even less evidence supporting the use of activity trackers to target sitting and standing: the activities that are most common in the office workplace setting [38,39]. As recently highlighted in a review [6], interventions that focus on increasing only physical activity do not necessarily result in changes in sitting; likewise, activity trackers that focus on steps and activity may not necessarily elicit changes in sitting time.

As such, the current study will pilot a waist-worn activity tracker (the LUMOback) that measures and notifies wearers of their sitting time, and also measures activities across the spectrum including number of steps and time spent standing, walking, and running [54]. Given the importance of organizational-level strategies [1,30], and the evidence suggesting self-monitoring tools are effective with additional strategies [42,53,55], the activity tracker will be implemented in conjunction with organizational-level support strategies. To evaluate whether the intervention strategies can also be sustained long-term, assessments will occur in the short (3 months) and long-term (12 months).

### Aims

The primary aims of this pilot study are to assess two interventions (organizational-level strategies only; organizational-level strategies plus an activity tracker) in office workers regarding their feasibility, acceptability, and short-term (3 month) effectiveness for sitting reduction. The primary effectiveness outcomes are sitting time at work and overall. Secondary aims are to examine the short-term effectiveness of the interventions for other activities, and health- and work-related outcomes; the relative effectiveness of the two interventions for changes in sitting time and other activities; predictors of changes in sitting and activity; and the long-term (12 month) feasibility, acceptability, and effectiveness of the interventions.

## Methods

### Design

**Figure 1:**
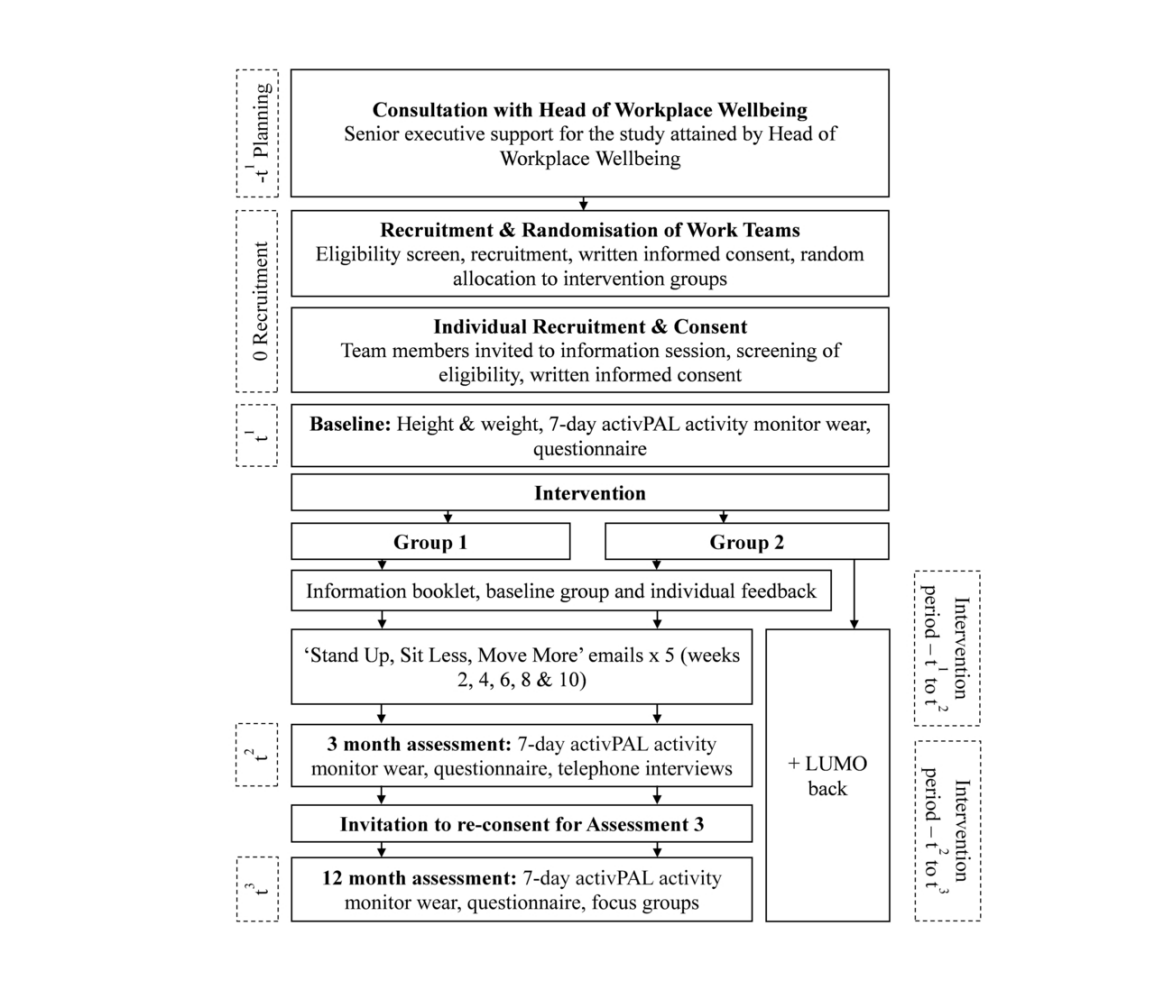
Overview of study design, consent processes, intervention, and assessment elements.

A cluster-randomized design will be used, with data collection occurring at baseline, 3, and 12 months (see Figure 1). The cluster-randomized design (with work teams as the unit of clustering) was chosen to minimize contamination among participants from the two intervention groups. The trial has been approved by the University of Queensland Behavioural and Social Sciences Ethical Review Committee (approval number: 2014000089) and is prospectively registered with the Australian New Zealand Clinical Trials Registry [registration number: ACTRN12614000252617].

### Participants

Participants will be office workers, recruited from work teams from two Australian capital cities (~1000-km apart) within the one organization, Lendlease, an international property and infrastructure group. Inclusion criteria are office-based workers, working at least 0.6 full-time equivalent (ie, at least 60% of full-time work hours) and ambulatory (able to walk at least 10 meters). Exclusion criteria are pregnancy at baseline, allergies to adhesive tape (Opsite and Hypafix required for assessments), and a planned absence from work for longer than 2 weeks during the first 3-month study period. Employees who have their own activity-permissive workstation at the baseline assessment will also be ineligible.

### Recruitment

#### Recruitment of Organization

The study liaison and workplace champion for the trial will be the Lendlease Head of Workplace Wellbeing (DCY). The workplace champion, located in Sydney (site A), will work in partnership with the research team to tailor the intervention to Lendlease and to create strong buy-in from senior management. Project staff will be based in Brisbane (site B).

#### Recruitment of Managers and Teams

Workplace teams (typically with 10-15 individuals in each team) will be selected from site A and site B by the workplace champion. Teams will be defined as having a line manager, being physically colocated, and having regular group meetings. Eligible teams will need to be desk-based and have a sufficient number of team members with access to a Bluetooth-enabled mobile phone required for the activity tracker to function. Team managers will be approached by the workplace champion for their consent.

#### Recruitment of Participants

Once the team manager has consented, the workplace champion will email individual team members the information sheet detailing the study and the required level of commitment, along with the consent form. Teams randomized to receive the activity tracker will receive additional details regarding the LUMOback. Interested team members will be invited to attend a face-to-face information session where they will have their eligibility assessed by project staff, and then be invited to sign the consent form and proceed to the baseline assessment. Initial consent will only be for baseline and 3-month assessments; participants will be invited to reconsent for the 12-month assessment (see Figure 1).

### Randomization

Following manager consent, and prior to the information session, each team will be numbered randomly, using a random number generator, and then listed in numeric order. Teams will then be randomized to either Group 1 (organizational-level strategies only) or Group 2 (organizational-level strategies plus activity tracker), across location strata (site A and B) and team size strata within site A (small/large) using a randomization website [56]. The randomization schedule will be created by a university staff member not involved in the study. A project staff member will then apply the randomization schedule to the list of teams. Neither project staff nor participants will be blinded to participants’ randomization condition.

### Organizational-Level Intervention

All participants will receive the organizational-support intervention elements, which are based on the *Stand Up Australia* intervention [57]. The key intervention message is ‘Stand Up, Sit Less, Move More’ [57], which encourages staff to interrupt sitting at least every 30 minutes with a change in posture; replace sitting with standing or moving (working toward having equal amounts of sitting and upright activities in the day); increase physical activity of any intensity level; and to make these changes across the whole day, both during and outside of work hours.

#### Information Booklet and Participant Feedback

All participants will receive a booklet from the workplace champion, developed by the research team and customized to Lendlease’s branding and corporate style requirements. The booklet will cover the study rationale (ie, evidence on prolonged sitting and detrimental health outcomes) and purpose; general guidelines on optimal workplace activity; behavior change strategies related to the key intervention messages; and general information about the study procedure and timeline. All participants will also receive a summary email from the workplace champion of the aggregate results from the baseline assessment regarding sitting, standing and stepping, as well as individual feedback on sitting, standing and stepping time after each of the assessment points (to be emailed individually by project staff; adapted from a previous study [39]).

#### Stand Up, Sit Less, Move More Emails

The workplace champion will create five emails in consultation with project staff (adapted from a previous study [58]). One email will be sent every 2 weeks during the initial 3-month intervention period. The emails will consist of a ‘tip of the week,’ a study update from the workplace champion, a quote from a participant and/or manager, and an infographic or a picture of Lendlease staff engaging in the ‘Stand Up, Sit Less, Move More’ message. Project staff will be included on the emails to enable tracking of email content for process evaluation. While the emails will cease after week 10, it is expected that team managers will continue to implement the strategies promoted in the emails with their team throughout the rest of the 12-month intervention period.

#### Executive Management Support

In addition to the staff, managers and senior managers participating in the trial, senior global executives (eg, Chief Executive Officer) will also take part in the baseline assessment and will receive the information booklet and ‘Stand Up, Sit Less, Move More’ emails. The participation of the global executives, which demonstrates visible support for the intervention, will be communicated to staff by the workplace champion.

### Activity Tracker

**Figure 2:**
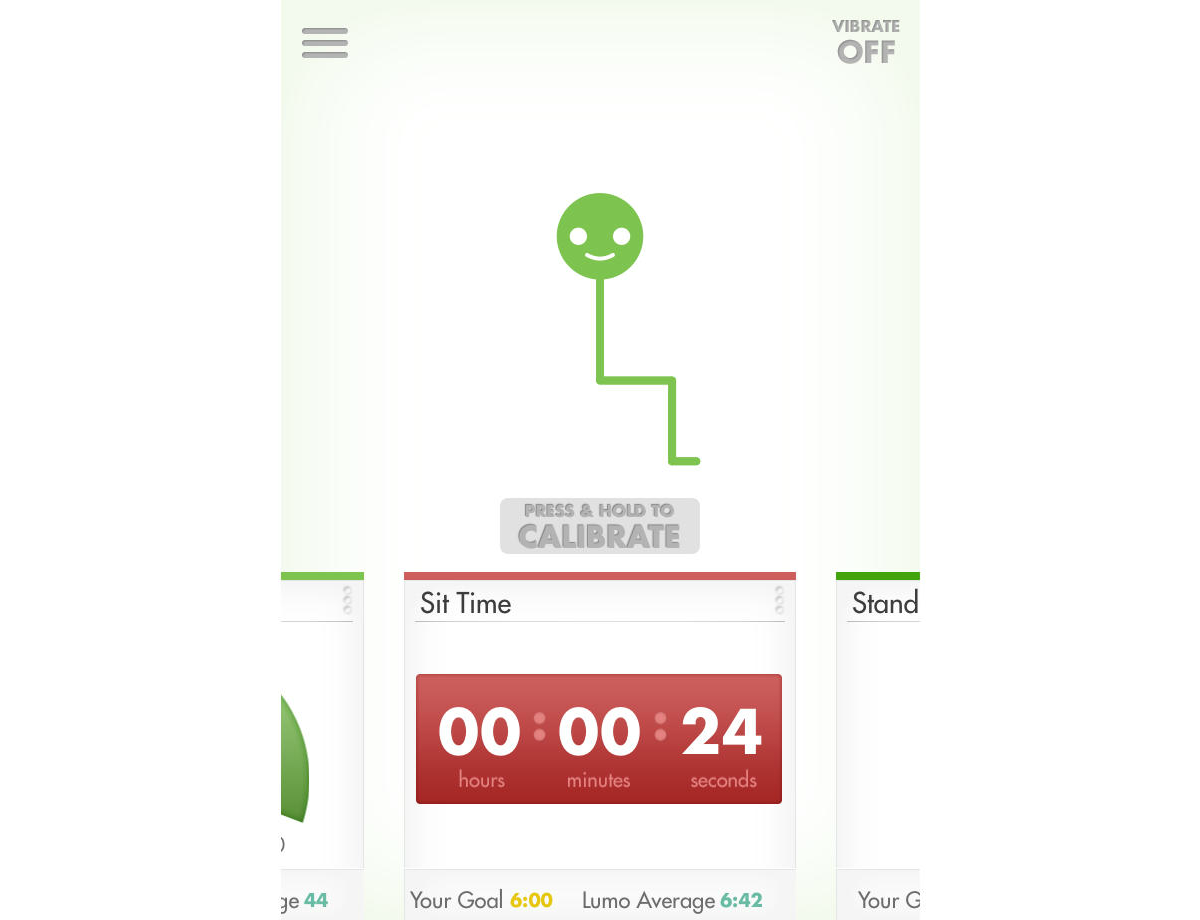
Representation of the LUMOback avatar while seated in a good posture.

Participants in Group 2 will be given the LUMOback activity tracker in addition to the organizational-level strategies. The LUMOback is worn around the waist as a belt, collecting information, and providing real-time feedback on sitting, standing, stepping, breaks from sitting, posture, and sleep. The LUMOback assesses activity by inertial sensors, which collect data at a constant 25 Hz [59], and is controlled through a mobile app via a Bluetooth connection that can be used by both iPhone operating system and Android platforms. Up to 3 weeks of data can be collected by the LUMOback before it must be synced with the app, with data transferred between the LUMOback and the app at 600 bytes/second. In the app, participants can view graphs, averages, and goals related to their sitting, standing, stepping, sitting breaks, posture (represented by an avatar, see Figure 2), and sleep. The device can monitor behavior, track attainment of the wearer’s goals, and provide real-time feedback. Participants can use the app to select the LUMOback to vibrate when they are sitting or standing in a ‘poor’ lumbar posture as identified by pelvic tilt angle [59], and to send a push notification to their mobile phone when they have been sitting too long. Vibrations can be chosen to be more or less intense, more or less exact about posture, or turned off; sitting notifications can be selected to range in time between 15 minutes and 2 hours of sitting, or turned off. The app also contains many learning videos about maintaining a good posture.

The LUMOback shows strong correlations and good agreement, in free-living conditions, with measures from the activPAL activity monitor in total time spent sitting (*R* ^2^=0.89 [60]; mean absolute percent error (MAPE)=9.5% [61]), standing (*R* ^2^=0.86 [60]), and number of steps (MAPE=0.4%, intraclass correlation coefficient (ICC)=0.99 [62]). In laboratory conditions, the LUMOback shows excellent agreement in step counts when tested against the Optogait treadmill test (MAPE=0.2%, ICC=0.99 [62]).

A LUMOback and a four-page instruction booklet that covers an introduction to the device, set-up instructions, and frequently asked questions will be distributed to Group 2 participants by the workplace champion following baseline assessments. On receipt of the device, participants will be asked to download the free app and sync the LUMOback with their phone. Participants will be asked to use their work email to set up their device as this will later be used to request the data from the company LUMO Bodytech. Participants can wear the device as much or as little as they like and their self-directed usage will be tracked. However, participants will be instructed by project staff to wear their LUMOback during the 3-month assessment period both to gather validity data and to estimate the intervention effectiveness while wearing the LUMOback. Participants will be allowed to keep the LUMOback.

### Data Collection

Data collection will occur at baseline, 3, and 12 months, and has been approved by the organization to occur during work hours. At each assessment, participants will be asked to wear an activPAL3 activity monitor continuously (24 hours/day) for 7 days, and to complete a concurrent electronic work and sleep diary. They will also be emailed a link to a Web-based questionnaire, using LimeService [63] covering demographic information (baseline assessment only), health- and work-related information (all assessments), and feasibility and acceptability (3- and 12-month assessments). Height and weight will be measured by a trained Lendlease staff member (site A) or project staff member (site B) during a face-to-face baseline assessment session that is expected to take approximately 15 minutes.

Individual qualitative interviews on the feasibility and acceptability of both of the interventions will occur following the 3-month assessment via telephone. Focus groups covering the long-term feasibility and acceptability of the interventions will be conducted as part of the 12-month assessment. Project staff will request LUMOback usage data from Lumo Bodytech every 2 weeks during the initial 3-month intervention period and then periodically throughout the rest of the study.

### Measures

An overview of the study feasibility, acceptability, and effectiveness outcome measures is provided in Table 1. In addition, a range of measures covering demographics, job, and health characteristics, psychosocial variables and technology confidence will also be collected.

#### Feasibility

Feasibility information will be examined in terms of participation, retention rates, and intervention delivery indicators, including the number of emails received (for the organizational intervention) and the degree of usage of the LUMOback activity tracker and app (assessed from the device and self-report). Ease of LUMOback data download and amount of lost or missing LUMOback data will also be evaluated. Table 1 displays an overview of the feasibility indicators and Multimedia Appendix 1 contains the questionnaire items created for the study.

The LUMOback data will be used to determine number of days, hours, and peak levels of usage. The data is expressed as a percentage of a 5-minute window spent in each activity type (sitting and standing in a good or bad posture, lying, sitting in a car, walking, and running), and step counts, calorie counts, and not worn/charging. No data is recorded over periods the device self-registers as off.

Telephone interviews and focus groups will be used to further evaluate both organizational support and LUMOback intervention strategies. Telephone interviews will identify: strategies implemented by the managers and organization; key facilitators and barriers of the intervention (including those specific to the LUMOback); and, any organizational culture changes that occurred (see Multimedia Appendix 2 for questions). Focus groups will discuss the impact of the interventions on the workplace culture and environment, long-term barriers and facilitators of the intervention strategies, and will identify any other key themes (see Multimedia Appendix 3).

#### Acceptability

Participants will be asked via questionnaire to rate how useful the emails and tips were, their satisfaction with the emails and information, and if they had experienced any discomfort or injury as a result of their study participation. Participants who indicate they have used a LUMOback device will also be asked to rate their comfort, ease of use, and perceived usefulness of the LUMOback (see Multimedia Appendix 1 for full list of questionnaire items). Participants who indicate they were given a LUMOback (whether they wore it or not) will also be asked would they recommend the LUMOback to a friend, how likely they were to use the LUMOback in the next 6 months, did they have any adverse experiences from using the LUMOback, and if they had any comments about the LUMOback or app. A shorter questionnaire at 12-month follow-up (to reduce participant burden and maintain compliance) will collect information relating to long-term LUMOback usage, adverse experiences, and comments. Telephone interviews and focus groups will also assess the acceptability of both interventions over the short and long-term (see Multimedia Appendices 2 and for questions).

#### Effectiveness

##### Activity Outcomes

The activPAL3 activity monitor will be used to measure the primary and secondary activity outcomes. The activPAL is a small device worn on the thigh (53×35×7 mm; 15 g). It has excellent interdevice reliability (ICC=0.79-0.99 [64]). It shows excellent validity relative to direct observation in measuring sitting/lying, hereafter termed ‘sitting,’ standing, and stepping, and for alterations between sitting and upright posture [64-66]. The activPAL has also shown responsiveness to change in sitting [66]. Correlations with direct observation of total time spent sitting, standing, and stepping, and total number of postural transitions are close to 1, mean differences reported are small and nonsignificant (eg, sitting and breaks measures all <2% bias) [65]. The agreement with direct observation of activity classification (sitting, standing, or stepping) moment by moment is also excellent (eg, 98.5% correct in a controlled setting, 93.6% correct during activities of daily living) [64]. Also, participants cannot extract activity data (real-time or otherwise) from the activPAL3, limiting reactivity.

The primary activity outcomes are time spent sitting at work and time spent sitting overall (ie, across all waking hours on work and nonwork days). The other activity outcomes relating to the ‘Stand Up, Sit Less, Move More’ message are listed in Table 1. In line with the exploratory nature of the pilot study, a range of other sedentary and physical activity measures (eg, usual sitting bout duration, number of steps) will be assessed and reported in addition to the study outcomes.

##### Health Outcomes

Physical and mental health-related quality of life [67] will be measured by the Short Form (SF)-12 version 1 at each assessment. The SF-12 correlates highly with the SF-36 (*r*=0.95 and 0.97) and has good test-retest scores (*r*=0.89 and 0.76) for the physical and mental components respectively [67]. Overall stress will be measured at each assessment by one item from the Health and Work Questionnaire [68].

##### Work Outcomes

Work-related outcomes will be measured via questionnaire at all assessments. These are work performance [69] and two measures from the Health and Work Questionnaire [68]: work satisfaction (four items, with α for internal consistency=0.84), and job control (one item).

**Table 1:** Overview of study outcomes.

Measurement tools	Outcomes
Feasibility
Organizational support questionnaire items	Number of emails received
	What participants did with email (eg, read then delete)
	Perceived level of support to ‘Stand Up, Sit Less and Move More’ from (1) organization, (2) main manager, and (3) colleagues
LUMOback questionnaire items	Ever used (eg, yes, given but never used, no)
	Date of first use
	Setting of use (eg, workplace)
	Frequency of recalibration, app checking, goal checking, vibration and sitting alerts
LUMOback data	Days of usage
	Hours of usage/day
	Peak levels of usage
	Ease of data download
	Amount of missing data
Telephone interviews	Strategies implemented by manager and organization, organizational culture change, key elements of intervention, barriers of wearing the LUMOback at work and at home
Focus groups	Long term sustainability of changes, barriers and facilitators, key themes
Acceptability
Organizational support questionnaire items	Usefulness of emails and tips
	Satisfaction with emails and information received
	Adverse experiences from program in general
LUMOback questionnaire items	Comfort of LUMOback
	Ease of set up of LUMOback and app, navigating app, calibrating LUMOback
	Usefulness of LUMOback and app
	Likelihood of using LUMOback in next 6 months, adverse experiences from LUMOback, comments about the LUMOback
Telephone interviews	Acceptability of program, acceptability of the LUMOback
Focus groups	Acceptability of program, acceptability of long-term LUMOback wear
Effectiveness
Activity
activPAL	Stand up: standing time at work and overall
	Sit less: sitting time at work and overall (primary effectiveness outcomes); prolonged (>=30 mins) sitting time at work and overall
	Move more: stepping time at work and overall
Health
SF-12 v1	Physical and Mental health-related quality of life	
Health and Work Questionnaire	Overall stress
Work
Work performance rating scale	Work performance
Health and Work Questionnaire	Work satisfaction, job control

#### Demographics and Job Characteristics

Height and weight will be measured with shoes removed using a stadiometer (to the nearest 0.1 cm) and calibrated electronic scales (to the nearest 0.1 kg) at baseline only. Similarly, participants will be asked at baseline only their age, gender, Aboriginal or Torres Strait Islander status, highest level of education completed, and length of time at the workplace. Pregnancy status will be measured at 3- and 12-month follow-ups only, while current smoking status and smoking while at work will be measured at each assessment.

Full-time equivalence, job category, and percent of work time spent at desk, away from desk, and outside the workplace [58] will be measured at all assessments. Frequency and duration of working with colleagues [58] will be assessed at baseline and 3-month follow-up only. At 3- and 12-month follow-ups, workplace location will be assessed to account for any office relocations. Data on team location and team size will be collected by project staff at baseline.

#### Other Measures

Several additional individual, work, health, and intervention factors will be assessed to explore whether they predict changes in sitting and activity, and for consideration as potential confounders for relative effectiveness. The additional individual factors to be assessed include: confidence with technology and use of any other apps or wearable devices to help increase activity (see Multimedia Appendix 1), as well as psychosocial measures such as preference for sitting and standing at work, and knowledge of the health impacts of sitting [58]. These psychosocial variables have been derived from previous *Stand Up Australia* research [58], have moderate to good test-retest reliability (Spearman’s ρ=0.67-0.78) [38,58], and will be assessed at baseline and 3-month follow-up only. The additional health-related factors include perceived stress and musculoskeletal health. Specifically, at baseline and 3-month assessments only, perceived stress will be measured using the 4-item version of the Perceived Stress Scale [70]. The 4-item Perceived Stress Scale has good internal consistency (α=0.82) and correlates moderately with the Impact of Event Scale (*r*=0.58), 12-item Posttraumatic Stress-Arousal Scale (*r*=0.70), and the mental health component of the Medical outcomes Scale-SF 36 (*r*=0.70) [71]. Musculoskeletal health will be measured at each assessment using a modified 36-item version of the Nordic Musculoskeletal Questionnaire [72]. Modifications are that items will refer to the last 1 month (instead of 12 months) [73] and items will be added to assess how intense the pain was in the body part in the last 1 month (on a 0-9 scale, where 0 means no complaints and 9 means pain as bad as it can be) for those who indicated that they experienced a problem [74] (questions can be found in Multimedia Appendix 1). Work factors such as perception of supervisor relations (two items from the Health and Work Questionnaire [68], with α for internal consistency=0.85) will be measured at each assessment. Use of workplace strategies to sit less and move more (adapted from *Stand Up Australia* [58], full list of questions Multimedia Appendix 1) will be measured at all three assessments. Self-report activity will also be collected via the Occupational Sitting and Physical Activity Questionnaire (OSPAQ) [75]. Assessment of sitting using the OSPAQ correlates moderately with accelerometer (Actigraph GT1M) measured sedentary time (Spearman ρ=0.65) [75].

### Assessment Procedures

#### activPAL Procedure

At baseline, the activPAL monitors and required adhesive materials (several Hypafix patches and alcohol swabs) will be provided to participants during a face-to-face assessment session. An in-person demonstration will be given at this session on how to wear the device (ie, on the dominant thigh on the midline, approximately one-third of the way down between the hip and the knee, attached using hypoallergenic adhesive material (Hypafix)). Participants will be asked to wear the monitor for seven consecutive days, 24 hours per day, removing only in circumstances during which the monitor is likely to be lost or damaged, but not for routine showering/bathing or swimming, as monitors will be waterproofed (with latex finger cots and hypoallergenic Opsite). Instructions will also be sent by email. Participants not able to attend the face-to-face baseline assessment session will receive an activPAL from the workplace champion after the session. At follow-up assessments, the activPALs will be distributed to participants by the workplace champion (site A) and either the workplace champion or by project staff to participants at site B. Site A participants will be instructed to return their monitor in sealed packs to the workplace champion, who will post these packs to project staff for download and processing. Site B assessment packs will be collected in person by project staff.

Concurrent diaries, covering waking hours, periods wearing/removing the activPAL, work days and times that are similar to previous paper-based versions [39,58] will be piloted in electronic forms (via LimeService and via a macroenabled excel-based file), with paper versions provided as an alternative option for those experiencing any difficulties with the electronic versions. The diary for Group 2 participants at 3-month follow-up will also cover LUMOback wear/removals, frequency of checking the LUMOback app, usage, and setting type of the vibrations and sitting time alerts, and usage of goal setting over the 7-day assessment period. Participants who do not complete all aspects of their diary will be recontacted to provide further details.

#### activPAL Data Processing

The measures for activity overall and activity during work hours will be extracted from the events-based activPAL data using procedures similar to a previous study [58] via a customized SAS program (version ≥9.3). Activity recorded by the activPAL during relevant periods (eg, working, awake, and wearing the monitor) will be ascertained from a combination of the diary information and the participant’s movement as recorded by the activPAL. When wear/waking hours are not reported, these will be inferred from the movement data. Bouts of activity will be assigned the classification (eg, awake/not, working/not) that applies to most (≥50%) of the bout. Sleeping periods will be adjusted to exclude any short bouts (<20 minutes duration) at the beginning or end of the sleeping period. Studies use a variety of definitions for ‘valid’ days of activPAL data [76]. Days will be considered valid for activity at work if the activPAL was worn for ≥80% of the time at work; entire waking days will be considered valid for activity if the activPAL is worn for ≥80% of waking hours and for ≥10 hours if waking hours are inferred from the activPAL rather than reported by the participant. Time spent in each activity will be calculated for each day then averaged over the valid days. Information about duration of bouts of activity (eg, usual sitting bout duration) will be calculated across all of the relevant time periods on valid days.

Quality control checks will be performed both prior to processing for the diary (missing information, nonconsecutive dates, activities finishing prior to starting, short waking days <10 hours), and postprocessing. The processed data will be checked visually (heatmaps) to verify the activity patterns were consistent with the classifications of the data as included (waking wear on valid days) or excluded (removal, sleep, or invalid days) and data will be reprocessed when errors are identified.

#### Questionnaire Procedure

At baseline, 3-, and 12-month assessments participants will be emailed a link to a Web-based questionnaire (LimeService) by project staff after they have finished wearing the activPAL. At baseline, questionnaires are to be completed before the intervention begins. At all stages, participants will be provided with the opportunity to opt out of the questionnaire.

#### Qualitative Interview and Focus Group Procedures

Semistructured telephone interviews will be conducted at the 3-month assessment. Attempts will be made to contact all participants in Group 2, with a similar number of participants recruited from Group 1, sampling purposively for diversity, starting with the two most disparate team members per team on age, gender, job category, and sitting time change. All team managers will also be approached for a telephone interview. All interviews will include questions to evaluate the organizational support intervention; Group 2 interviews will also assess the LUMOback. Interviews will be recorded using Audacity (version 2.0.6) and transcribed verbatim with idiosyncratic elements of speech removed. All participants who remain in the study at 12-month follow-up will be invited to take part in focus group interviews, which will be capped at 10 participants each. Participants will be offered a chance to win an activity tracker (single prize; randomized prize draw; value ~AU$500) for participating in the focus groups. Focus group interviews will be audio-recorded and transcribed.

### Sample Size

For this pilot study, the sample size was selected based on what the workplace deemed feasible (18 teams). With a usual team size of 10 to 15, and two-thirds expected to be eligible and participate (just over eight per team), we anticipate approximately 150 participants in total, with 75 randomized to each group. This sample size will provide adequate power (≥80% power) with 5% two-tailed significance, to detect short term (3 month) changes within groups (primary effectiveness aim) of our minimum difference of interest (MDI) in our primary outcomes of work and overall sitting time (see Table 2). All calculations are based on no multiple comparison adjustment to significance (in line with the exploratory nature of the study), an anticipated 30% attrition, and strong clustering (ICC=0.1) [58] with an anticipated design effect of 1.48 (ie, 1+0.1×4.83, with an average 5.83 participants per team). Under these same assumptions, power to detect changes equal to the MDIs for the secondary activity outcomes with 5% two-tailed significance are presented in Table 2 , along with the minimum detectable differences (MDDs) between groups for relative effectiveness (secondary aim). This pilot did not power a priori on other research questions, such as health outcomes and long-term changes (12 months). The MDIs and MDDs all reflected modest effects for activity. The assumptions regarding standard deviations (SD), pre-post correlations and clustering were informed by published and unpublished data from previous workplace interventions [38,58] and Australian population data from the AusDiab study.

**Table 2:** Power to detect changes within groups (effectiveness) and minimum detectable differences between groups (relative effectiveness) with 5% significance, two-tailed.

Outcome	MDI^a^	Assumed values		Effectiveness	Relative effectiveness
		SD^b,c^	Pre-post *r* ^c^	Power	MDD^d^
Primary outcomes					
Work sitting time	45 minutes	90	0.6	90%	50 minutes
Overall sitting time	45 minutes	90	0.6	90%	50 minutes
Secondary outcomes					
Work prolonged sitting time	45 minutes	120	0.6	67%	65 minutes
Overall prolonged sitting time	45 minutes	120	0.6	67%	65 minutes
Work standing time	30 minutes	70	0.6	79%	35 minutes
Overall standing time	30 minutes	70	0.6	79%	35 minutes
Work stepping time	15 minutes	20	0.7	>99%	10 minutes
Overall stepping time	15 minutes	30	0.7	96%	15 minutes

^a^minimum difference of interest.

^b^standard deviation.

^c^assumed values based on unpublished data from the *Stand Up Victoria* trial

^d^minimum detectable difference with 80% power

### Statistical Analyses

Statistical analyses will be conducted in SPSS Statistics version ≥22 and Stata version ≥13 with statistical significance set at *P* <.05, two-tailed. Within-group changes in activity, work and health outcomes (continuous) will be assessed, using linear mixed-models that account for repeated measures and clustering, to determine the effectiveness of each intervention for these outcomes in the short- and long-term. To compare the relative effectiveness of the combined organizational-level and activity tracker intervention to organizational support alone, mixed-models will be used, adjusting for baseline values and potential confounders; these address both repeated measures and clustering. Confounders will initially be chosen a priori from the literature and retained in models if they are associated with the outcome at *P* <.2. Models will be checked for linearity, normality, and heteroscedasticity. Analyses will follow intention-to-treat principles. Per-protocol analyses will also be conducted to evaluate what the efficacy of the intervention is specifically for those who use the activity tracker, because activity tracker usage is self-directed. Assumptions regarding missing data will be checked and sensitivity analyses will be conducted to evaluate the impact of missing data on findings. Predictors of changes in activity will be evaluated by linear regression, adjusting for baseline values, and correcting for clustering. Predictors will be considered separately and also mutually adjusted. Characteristics of individuals will be considered as potential predictors including demographics (eg, age), psychosocial (eg, preference for sitting at work), health (eg, musculoskeletal problems) and work-related characteristics (eg, perception of relationship with supervisor), and engagement with the intervention (eg, LUMOback usage).

#### Feasibility and Acceptability

Feasibility and acceptability data acquired by questionnaire, LUMOback data, and participation and retention rates will be reported using descriptive statistics. Content analyses in NVivo (version 10) will be conducted with the telephone interviews to derive reception toward both interventions, barriers and facilitators, and any other themes. Data from the focus groups will be thematically analyzed by two independent authors and discussed with a third author. Any discrepancies in themes will be discussed until consensus is reached.

## Results

Baseline and follow up data collection has finished. Results are expected in 2016.

## Discussion

This paper describes the background, design, and methods of a pilot, cluster-randomized trial that will compare two interventions, one that targets organizational-level strategies and another that targets organizational-level strategies plus the use of a wearable activity tracker, the LUMOback, in office workers. The study will determine if either intervention can produce changes in sitting (during all waking hours and work hours), as well as prolonged sitting, standing, and stepping in the short (3 month) and long-term (12 month), as well as the feasibility and acceptability of each intervention. The impact of each intervention on health- and work-related outcomes, and the predictors of sitting and activity change will also be examined. In addition, the study will provide preliminary evidence regarding the additional impact of the LUMOback on sitting and activity compared with organizational-level strategies alone.

The interventions in this study are designed to be easily disseminated on a large scale. Specifically, the intervention elements will come from the Head of Workplace Wellbeing, making the implementation of the intervention similar to that which may realistically occur in office workplaces. The intervention messages are delivered via a low cost, feasible mechanism (emails), with the LUMOback device also being relatively low cost, and comparable in price with other popular fitness trackers on the market (~US$150). Another strength of the intervention is that support and participation will come from multiple levels of the organization (ie, general staff, managers, senior global executives). In addition, sitting, standing and stepping will be measured at and away from the workplace, with a validated and objective measure. Many workplace studies have only measured workplace activity, and have often not used objective, posture-based measures.

### Methodological Considerations

Because we will work in partnership with the organization, it will not be possible to recruit a control group who will not receive the organizational intervention. As such, the effectiveness of each intervention can only be evaluated as per a single-group pre–post design. Accordingly, effectiveness findings will be considered in light of the usual findings within other studies’ control groups. For example, in prior *Stand Up Australia* research [38,39], there were no significant changes in sitting, standing, and stepping within control groups. Another consideration is contamination. Despite randomization occurring at the team level to reduce contamination, we will not know in advance the degree of interaction between the teams, and therefore the extent to which the participants randomized to Group 1 may receive visual cues to stand up by witnessing LUMOback wearers (Group 2) arise in response to device prompting. Focus groups will attempt to evaluate if any potential contamination occurred. In addition, another consideration is that the Head of Workplace Wellbeing will select the teams for participation. While this is not inconsistent with what might realistically occur in office wellness programs, it may introduce some selection bias, which may limit the generalizability of the results.

### Conclusions

The interventions evaluated in this study have the potential to decrease sitting and increase standing and stepping in the office workplace. There has been minimal evaluation of organizational-level strategies alone, and whether these strategies can impact on sitting and activity behaviors when delivered as part of a worksite driven, ‘real-world’ intervention. Furthermore, there is minimal evidence on the feasibility, acceptability, and effectiveness of activity trackers for use in office workers and their effectiveness for reducing sitting and increasing standing and stepping. Despite the detrimental effects of sitting, very few activity trackers measure or target this behavior. If effective, the findings from this research may prompt developers to include sitting measures and prompts in their activity trackers. While only a pilot, this study aims to address these gaps and will provide information to guide future physical activity and sedentary behavior interventions and workplace health promotion programs.

## Appendix

Multimedia Appendix 1 Questionnaire items created or modified for study.

Multimedia Appendix 2 Telephone interview questions.

Multimedia Appendix 3 Focus group questions.

## Abbreviations

ICC: intraclass correlation coefficient
MAPE: mean absolute percent error
MDD: minimum detectable difference
MDI: minimum difference of interest
METS: metabolic equivalents
MVPA: moderate- and vigorous-intensity physical activities
OSPAQ: Occupational Sitting and Physical Activity Questionnaire
SD: standard deviation
SF: short form

## References

[1] Burton J (2010). World Health Organization.

[2] Goetzel RZ, Ozminkowski RJ (2008). The health and cost benefits of work site health-promotion programs. Annu Rev Public Health.

[3] Conn VS, Hafdahl AR, Cooper PS, Brown LM, Lusk SL (2009). Meta-analysis of workplace physical activity interventions. Am J Prev Med.

[4] Marshall AL (2004). Challenges and opportunities for promoting physical activity in the workplace. J Sci Med Sport.

[5] Chau JY, van der Ploeg HP, van Uffelen JGZ, Wong J, Riphagen I, Healy GN, Gilson ND, Dunstan DW, Bauman AE, Owen N, Brown WJ (2010). Are workplace interventions to reduce sitting effective? A systematic review. Prev Med.

[6] Martin A, Fitzsimons C, Jepson R, Saunders DH, van der Ploeg HP, Teixeira PJ, Gray CM, Mutrie N, EuroFIT consortium (2015). Interventions with potential to reduce sedentary time in adults: systematic review and meta-analysis. Br J Sports Med.

[7] Sedentary Behaviour Research Network (2012). Letter to the editor: standardized use of the terms "sedentary" and "sedentary behaviours". Appl Physiol Nutr Metab.

[8] Norton K, Norton L, Sadgrove D (2010). Position statement on physical activity and exercise intensity terminology. J Sci Med Sport.

[9] Hamilton MT, Healy GN, Dunstan DW, Zderic TW, Owen N (2008). Too little exercise and too much sitting: inactivity physiology and the need for new recommendations on sedentary behavior. Curr Cardiovasc Risk Rep.

[10] Straker L, Healy GN, Atherton R, Dunstan DW (2014). Excessive occupational sitting is not a "safe system of work": time for doctors to get chatting with patients. Med J Aust.

[11] Healy GN, Dunstan DW, Salmon J, Cerin E, Shaw JE, Zimmet PZ, Owen N (2007). Objectively measured light-intensity physical activity is independently associated with 2-h plasma glucose. Diabetes Care.

[12] Buman MP, Hekler EB, Haskell WL, Pruitt L, Conway TL, Cain KL, Sallis JF, Saelens BE, Frank LD, King AC (2010). Objective light-intensity physical activity associations with rated health in older adults. Am J Epidemiol.

[13] Loprinzi PD (2013). Objectively measured light and moderate-to-vigorous physical activity is associated with lower depression levels among older US adults. Aging Ment Health.

[14] Thorp AA, Kingwell BA, English C, Hammond L, Sethi P, Owen N, Dunstan DW (2016). Alternating sitting and standing increases the workplace energy expenditure of overweight adults. J Phys Act Health.

[15] Healy GN, Winkler EAH, Owen N, Anuradha S, Dunstan DW (2015). Replacing sitting time with standing or stepping: associations with cardio-metabolic risk biomarkers. Eur Heart J.

[16] Wilmot EG, Edwardson CL, Achana FA, Davies MJ, Gorely T, Gray LJ, Khunti K, Yates T, Biddle SJ (2012). Sedentary time in adults and the association with diabetes, cardiovascular disease and death: systematic review and meta-analysis. Diabetologia.

[17] Biswas A, Oh PI, Faulkner GE, Bajaj RR, Silver MA, Mitchell MS, Alter DA (2015). Sedentary time and its association with risk for disease incidence, mortality, and hospitalization in adults: a systematic review and meta-analysis. Ann Intern Med.

[18] Brocklebank LA, Falconer CL, Page AS, Perry R, Cooper AR (2015). Accelerometer-measured sedentary time and cardiometabolic biomarkers: A systematic review. Prev Med.

[19] Healy GN, Dunstan DW, Salmon J, Cerin E, Shaw JE, Zimmet PZ, Owen N (2008). Breaks in sedentary time: beneficial associations with metabolic risk. Diabetes Care.

[20] Healy GN, Matthews CE, Dunstan DW, Winkler EAH, Owen N (2011). Sedentary time and cardio-metabolic biomarkers in US adults: NHANES 2003-06. Eur Heart J.

[21] Dunstan DW, Kingwell BA, Larsen R, Healy GN, Cerin E, Hamilton MT, Shaw JE, Bertovic DA, Zimmet PZ, Salmon J, Owen N (2012). Breaking up prolonged sitting reduces postprandial glucose and insulin responses. Diabetes Care.

[22] Peddie MC, Bone JL, Rehrer NJ, Skeaff CM, Gray AR, Perry TL (2013). Breaking prolonged sitting reduces postprandial glycemia in healthy, normal-weight adults: a randomized crossover trial. Am J Clin Nutr.

[23] Parry S, Straker L (2013). The contribution of office work to sedentary behaviour associated risk. BMC Public Health.

[24] Clemes SA, O'Connell SE, Edwardson CL (2014). Office workers' objectively measured sedentary behavior and physical activity during and outside working hours. J Occup Environ Med.

[25] Thorp AA, Healy GN, Winkler E, Clark BK, Gardiner PA, Owen N, Dunstan DW (2012). Prolonged sedentary time and physical activity in workplace and non-work contexts: a cross-sectional study of office, customer service and call centre employees. Int J Behav Nutr Phys Act.

[26] Ryan CG, Dall PM, Granat MH, Grant PM (2011). Sitting patterns at work: objective measurement of adherence to current recommendations. Ergonomics.

[27] Parry S, Straker L, Gilson ND, Smith AJ (2013). Participatory workplace interventions can reduce sedentary time for office workers--a randomised controlled trial. PLoS One.

[28] (2015). U.S. Bureau of Labor Statistics.

[29] Healy G, Lawler S, Thorp A, Neuhaus M, Robson E, Owen N, Dunstan D (2012). VicHealth.

[30] McLeroy KR, Bibeau D, Steckler A, Glanz K (1988). An ecological perspective on health promotion programs. Health Educ Q.

[31] Owen N, Sugiyama T, Eakin EE, Gardiner PA, Tremblay MS, Sallis JF (2011). Adults' sedentary behavior determinants and interventions. Am J Prev Med.

[32] Sallis JF, Cervero RB, Ascher W, Henderson KA, Kraft MK, Kerr J (2006). An ecological approach to creating active living communities. Annu Rev Public Health.

[33] Wierenga D, Engbers LH, Van EP, Duijts S, Hildebrandt VH, Van MW (2013). What is actually measured in process evaluations for worksite health promotion programs: a systematic review. BMC Public Health.

[34] Aldana SG, Anderson DR, Adams TB, Whitmer RW, Merrill RM, George V, Noyce J (2012). A review of the knowledge base on healthy worksite culture. J Occup Environ Med.

[35] Pronk N (2014). Best practice design principles of worksite health and wellness programs. ACSM’s Health & Fitness Journal.

[36] To QG, Chen TTL, Magnussen CG, To KG (2013). Workplace physical activity interventions: a systematic review. Am J Health Promot.

[37] Harden A, Peersman G, Oliver S, Mauthner M, Oakley A (1999). A systematic review of the effectiveness of health promotion interventions in the workplace. Occup Med (Lond).

[38] Healy GN, Eakin EG, Lamontagne AD, Owen N, Winkler EAH, Wiesner G, Gunning L, Neuhaus M, Lawler S, Fjeldsoe BS, Dunstan DW (2013). Reducing sitting time in office workers: short-term efficacy of a multicomponent intervention. Prev Med.

[39] Neuhaus M, Healy GN, Dunstan DW, Owen N, Eakin EG (2014). Workplace sitting and height-adjustable workstations: a randomized controlled trial. Am J Prev Med.

[40] Pronk NP, Katz AS, Lowry M, Payfer JR (2012). Reducing occupational sitting time and improving worker health: the Take-a-Stand Project, 2011. Prev Chronic Dis.

[41] Verweij LM, Proper KI, Leffelaar ER, Weel ANH, Nauta AP, Hulshof CTJ, van Mechelen W (2012). Barriers and facilitators to implementation of an occupational health guideline aimed at preventing weight gain among employees in the Netherlands. J Occup Environ Med.

[42] Dugdill L, Brettle A, Hulme C, McCluskey S, Long A (2008). Workplace physical activity interventions: a systematic review. Intl J of Workplace Health Mgt.

[43] Macniven R, Engelen L, Kacen MJ, Bauman A (2015). Does a corporate worksite physical activity program reach those who are inactive? Findings from an evaluation of the Global Corporate Challenge. Health Promot J Austr.

[44] Freak-Poli R, Wolfe R, Backholer K, de Courten M, Peeters A (2011). Impact of a pedometer-based workplace health program on cardiovascular and diabetes risk profile. Prev Med.

[45] Ribeiro MA, Martins MA, Carvalho CR (2014). Interventions to increase physical activity in middle-age women at the workplace: a randomized controlled trial. Med Sci Sports Exerc.

[46] Gilson ND, Faulkner G, Murphy MH, Meyer MR, Washington T, Ryde GC, Arbour-Nicitopoulos KP, Dillon KA (2013). Walk@Work: an automated intervention to increase walking in university employees not achieving 10,000 daily steps. Prev Med.

[47] Borg J, Merom D, Rissel C (2010). Staff walking program: a quasi-experimental trial of maintenance newsletters to maintain walking following a pedometer program. Health Promot J Austr.

[48] Faghri PD, Omokaro C, Parker C, Nichols E, Gustavesen S, Blozie E (2008). E-technology and pedometer walking program to increase physical activity at work. J Prim Prev.

[49] Lyons EJ, Lewis ZH, Mayrsohn BG, Rowland JL (2014). Behavior change techniques implemented in electronic lifestyle activity monitors: a systematic content analysis. J Med Internet Res.

[50] Fogg B (2002). Persuasive Technology: Using Computers to Change What We Think and Do.

[51] Zielinski D (2014). Wearable Wellness. HRMagazine.

[52] Nield D (2014). Fortune.

[53] Lewis ZH, Lyons EJ, Jarvis JM, Baillargeon J (2015). Using an electronic activity monitor system as an intervention modality: A systematic review. BMC Public Health.

[54] Lumo Bodytech.

[55] Evans L (2014). Entrepreneur.

[56] (2013). Randomization.com.

[57] Neuhaus M, Healy GN, Fjeldsoe BS, Lawler S, Owen N, Dunstan DW, LaMontagne AD, Eakin EG (2014). Iterative development of Stand Up Australia: a multi-component intervention to reduce workplace sitting. Int J Behav Nutr Phys Act.

[58] Dunstan DW, Wiesner G, Eakin EG, Neuhaus M, Owen N, LaMontagne AD, Moodie M, Winkler EAH, Fjeldsoe BS, Lawler S, Healy GN (2013). Reducing office workers' sitting time: rationale and study design for the Stand Up Victoria cluster randomized trial. BMC Public Health.

[59] Perkash R (2013). Lumo Bodytech.

[60] Rosenberger M, Haskell W, Buman M, LaStofka B, Carstensen L (2014). Abstract P128: a new device for objective measurement of sedentary behavior. Circulation.

[61] Rosenberger ME, Buman MP, Haskell WL, McConnell MV, Carstensen LL (2016). 24 hours of sleep, sedentary behavior, and physical activity with nine wearable devices. Med Sci Sports Exerc.

[62] Kooiman TJM, Dontje ML, Sprenger SR, Krijnen WP, van der Schans CP, de Groot M (2015). Reliability and validity of ten consumer activity trackers. BMC Sports Sci Med Rehabil.

[63] LimeService.

[64] Grant PM, Ryan CG, Tigbe WW, Granat MH (2006). The validation of a novel activity monitor in the measurement of posture and motion during everyday activities. Br J Sports Med.

[65] Lyden K, Kozey Keadle SL, Staudenmayer JW, Freedson PS (2012). Validity of two wearable monitors to estimate breaks from sedentary time. Med Sci Sports Exerc.

[66] Kozey-Keadle S, Libertine A, Lyden K, Staudenmayer J, Freedson PS (2011). Validation of wearable monitors for assessing sedentary behavior. Med Sci Sports Exerc.

[67] Ware J, Kosinski M, Keller SD (1996). A 12-Item Short-Form Health Survey: construction of scales and preliminary tests of reliability and validity. Med Care.

[68] Shikiar R, Halpern MT, Rentz AM, Khan ZM (2004). Development of the Health and Work Questionnaire (HWQ): an instrument for assessing workplace productivity in relation to worker health. Work.

[69] Sundstrom E, Town JP, Rice RW, Osborn DP, Brill M (1994). Office noise, satisfaction, and performance. Environ Behav.

[70] Cohen S, Kamarck T, Mermelstein R (1983). A global measure of perceived stress. J Health Soc Behav.

[71] Mitchell AM, Crane PA, Kim Y (2008). Perceived stress in survivors of suicide: psychometric properties of the Perceived Stress Scale. Res Nurs Health.

[72] Kuorinka I, Jonsson B, Kilbom A, Vinterberg H, Biering-Sørensen F, Andersson G, Jørgensen K (1987). Standardised Nordic questionnaires for the analysis of musculoskeletal symptoms. Appl Ergon.

[73] Kjaer P, Wedderkopp N, Korsholm L, Leboeuf-Yde C (2011). Prevalence and tracking of back pain from childhood to adolescence. BMC Musculoskelet Disord.

[74] Andersen LL, Clausen T, Burr H, Holtermann A (2012). Threshold of musculoskeletal pain intensity for increased risk of long-term sickness absence among female healthcare workers in eldercare. PLoS One.

[75] Chau JY, Van Der Ploeg HP, Dunn S, Kurko J, Bauman AE (2012). Validity of the occupational sitting and physical activity questionnaire. Med Sci Sports Exerc.

[76] Edwardson CL, Winkler EAH, Bodicoat DH, Yates T, Davies MJ, Dunstan DW, Healy GN (2016). Considerations when using the activPAL monitor in field based research with adult populations. J Sport Health Sci.

