# Supplementary material for: Organizational-Level Strategies With or Without an Activity Tracker to Reduce Office Workers’ Sitting Time: Rationale and Study Design of a Pilot Cluster-Randomized Trial
Source: JMIR Res Protoc. 2016 May 25;5(2):e73. doi: 10.2196/resprot.5438 (PMC4909392; doi:10.2196/resprot.5438)
Supplement: Multimedia Appendix 1 [file resprot_v5i2e73_app1.pdf]

Multimedia Appendix 1: questionnaire items created or modified for study

Organisational support program evaluation questions	Response options	Source
**How many Stand Up, Sit Less, Move More emails do you recall receiving from [the Head of Workplace Wellbeing] over the duration of the 'Stand Up Lend Lease' program?**	0-7 (indicates total number of emails)	Q 3M
**When you received a Stand Up, Sit Less, Move More email from [the Head of Workplace Wellbeing] what did you normally do with it?**	1 = Read it then delete it<br>2 = Read it then store it to look at later<br>3 = Leave it and not read it<br>4 = Delete it without reading it	Q 3M
**How supportive were the following parties at supporting you to Stand Up, Sit Less or Move More?**<br>• **your organisation?**<br>• **your main manager?**<br>• **your colleagues?**<br>***limited to the past 6 months for 12 month Q***	1 = Not supportive<br>2 = Somewhat supportive<br>3 = Not sure<br>4 = Supportive<br>5 = Extremely supportive	Q 3M, 12M
**How useful were the Stand Up, Sit Less, Move More emails and tips for helping you to:**<br>• **reduce your sitting**<br>• **increase your sit to stand transitions or "Stand Ups"**<br>• **increase your standing**<br>• **increase your step count**<br>• **increase your incidental activity**<br>• **increase your moderate to vigorous physical activity**	1 = Not at all useful<br>2 = Somewhat useful<br>3 = Not sure<br>4 = Useful<br>5 = Extremely useful	Q 3M
**Overall, how would you rate your satisfaction with the Stand Up, Sit Less, Move More emails and information you received?**	1 = Not at all satisfied<br>2 = Somewhat satisfied<br>3 = Not sure<br>4 = Satisfied<br>5 = Extremely satisfied	Q 3M
**Overall, how would you rate your satisfaction with**	Free text	Q 3M

**the Stand Up, Sit Less, Move More emails and information you received?**		
**Have you experienced any discomfort or injury as a result of your study participation?**	1 = Yes<br><br>2 = No	Q 3M, 12M
**Have you experienced any discomfort or injury as a result of your study participation?**	Free text	Q 3M, 12M
**Are there any other comments you would like to make about the 'Stand Up Lend Lease' program?**	Free text	Q 3M, 12M

Q = questionnaire; 3M = 3-month assessment; 12M = 12-month assessment

LUMOback evaluation questions	Response options	Source
**Have you ever used a LUMOback posture sensor device?**	1 = Yes<br>2 = No<br>3 = Given, but never used (asked at 3-months only)	Q BL, 3M, 12M
**When did you first start using the LUMOback posture sensor device?**	date	Q BL, 3M, 12M
**Please describe your usage of the LUMOback device in the past 6 months.**	1 = Have worn in the past week<br>2 = Have worn in the past month, but not in the past week<br>3 = Have worn in the past 6 months, but not in the past month<br>4 = Not worn in the past 6 months	Q 12M
**Did you ever wear your LUMOback:**<br>• **(1) at a Lend Lease workplace (includes your usual office and other Lend Lease sites)?**<br>• **(2) while working for Lend Lease from locations other than a Lend Lease workplace (such as from home or at an external conference)?**<br>• **(3) outside of work (e.g., commuting or in your leisure time)?**	1 = Yes<br>2 = No<br>3 = Not applicable [(2) only]	Q 3M
**Thinking about when you wore your LUMOback, how often did you wear your LUMOback:**<br>• **while at a Lend Lease workplace (includes your usual office and other Lend Lease sites)?**<br>• **while working for Lend Lease from locations other than a Lend Lease workplace (such as from home or at an external conference)?**<br>• **outside of work?**	1 = Rarely<br>2 = Sometimes<br>3 = Often	Q 3M
**What phone or device did you use the LUMOback with?**	1 = iPhone<br>2 = iPad<br>3 = Android phone<br>4 = N/A never set up LUMOback with phone or device	Q 3M

	5 = other	
**On days when you wore your LUMOback, how often did you check the LUMOback app on average?**<br><br>***limited to the past 6 months for 12 month Q**	1 = More than 10 times per day<br>2 = 5-10 times per day<br>3 = 2-4 times per day<br>4 = Once per day<br>5 = Less than once a day<br>6 = Never	Q 3M, 12M
**When using the LUMOback app, how often did you notice or check your goals? [goals include those set by you or the default goals created by LUMOback]**<br><br>***limited to the past 6 months for 12 month Q**	1 = Never<br>2 = Rarely<br>3 = Sometimes<br>4 = Often<br>5 = Very often<br>6 = All the time	Q 3M, 12M
**When wearing your LUMOback, how often did you re-calibrate the LUMOback device?**	1 = Never<br>2 = Rarely<br>3 = Sometimes<br>4 = Often<br>5 = Very often	Q 3M
**When wearing your LUMOback, how often did you: have it set to vibrate**<br><br>***limited to the past 6 months for 12 month Q**	1 = Never<br>2 = Rarely<br>3 = Sometimes<br>4 = Often<br>5 = Very often<br>6 = All the time	Q 3M, 12M
**When wearing your LUMOback, how often did you: have a sitting alert notification enabled**<br><br>***limited to the past 6 months for 12 month Q**	1 = Never<br>2 = Rarely<br>3 = Sometimes<br>4 = Often<br>5 = Very often<br>6 = All the time	Q 3M, 12M
**When you used the vibration feature did you mostly set the LUMOback to:**<br><br>• **pulse: the sensor buzzes gently until you move**	1 = Pulse<br>2 = Buzz once	Q 3M

**into good posture**<br>• **or to buzz once: the sensor vibrates once, each time you move into bad posture?**	3 = I used a combination<br>4 = Not sure	
**When you used the sitting alert notification did you mostly set it to alert every:**	1 = 15 mins<br>2 = 30 mins<br>3 = 45 mins<br>4 = 1 hr<br>5 = 2 hrs	Q 3M
**What learning videos did you watch in the app? (Please select all that apply)**	Sitting Posture<br>Standing Posture<br>Chest Stretch<br>Pec Stretch<br>Desk Prone Stretch<br>Pivot Prone Stretch<br>Cervical AROM Retraction<br>Did not watch any learning videos	Q 3M
**How comfortable was the LUMOback device to wear?**	1 = Not at all comfortable<br>2 = Somewhat comfortable<br>3 = Not sure<br>4 = Comfortable<br>5 = Extremely comfortable	Q 3M
**How easy was it to:**<br>• **set up the LUMOback device and app**<br>• **use and navigate the LUMOback app**<br>• **calibrate the LUMOback device**	1 = Not at all easy<br>2 = Somewhat easy<br>3 = Not sure<br>4 = Easy<br>5 = Extremely easy	Q 3M
**How useful were the LUMOback and the LUMOback app for helping you to:**<br>• **reduce your sitting**<br>• **increase your sit to stand transitions or "Stand Ups"**<br>• **increase your standing**	1 = Not at all useful<br>2 = Somewhat useful<br>3 = Not sure<br>4 = Useful<br>5 = Extremely useful	Q 3M

• **increase your step count**<br>• **improve your posture**<br>• **improve your sleep**<br>• **increase your incidental activity**<br>• **increase your moderate to vigorous physical activity**		
**How useful were the vibrations for:**<br>• **reducing your sitting**<br>• **making you aware of your posture**<br>• **motivating you to improve your posture**	1 = Not at all useful<br>2 = Somewhat useful<br>3 = Not sure<br>4 = Useful<br>5 = Extremely useful	Q 3M
**How useful were the sitting alerts for:**<br>• **making you aware of your sitting**<br>• **motivating you to reduce your sitting**<br>• **motivating you to increase your sit to stand transitions or "Stand Ups"**	1 = Not at all useful<br>2 = Somewhat useful<br>3 = Not sure<br>4 = Useful<br>5 = Extremely useful	Q 3M
**In the LUMOback app, how useful was the stick figure avatar at helping you to:**<br>• **correct your posture**<br>• **reduce your sitting**	1 = Not at all useful<br>2 = Somewhat useful<br>3 = Not sure<br>4 = Useful<br>5 = Extremely useful<br>6 = Did not notice/use	Q 3M
**In the LUMOback app, how useful were the goals for helping you to:**<br>• **reduce your sitting**<br>• **increase your sit to stand transitions or "Stand Ups"**<br>• **increase your step count**<br>• **improve your posture**	1 = Not at all useful<br>2 = Somewhat useful<br>3 = Not sure<br>4 = Useful<br>5 = Extremely useful<br>6 = Did not notice/use	Q 3M
**How satisfied did you feel when achieving either your own or the LUMOback goal?**	1 = Not at all satisfied<br>2 = Somewhat satisfied<br>3 = Not sure<br>4 = Satisfied	Q 3M

	5 = Extremely satisfied<br>6 = N/A never achieved any goals	
**How useful were your LUMOback graphs and results for making you aware of your:**<br>• **sitting**<br>• **sit to stand transitions or "Stand Ups"**<br>• **standing**<br>• **step count**<br>• **posture**	1 = Not at all useful<br>2 = Somewhat useful<br>3 = Not sure<br>4 = Useful<br>5 = Extremely useful<br>6 = Did not notice/use	Q 3M
**How motivating were your LUMOback graphs and results for:**<br>• **reducing your sitting**<br>• **increasing your sit to stand transitions or "Stand Ups"**<br>• **increasing your standing**<br>• **increasing your step count**<br>• **improving your posture**	1 = Not at all motivating<br>2 = Somewhat motivating<br>3 = Not sure<br>4 = Motivating<br>5 = Extremely motivating<br>6 = Did not notice/use	Q 3M
**When wearing your LUMOback how often did you:**<br>• **share or compare your LUMOback graphs and results with your co-workers?**<br>• **share your LUMOback graphs and results with your family or friends?**	1 = Never<br>2 = Rarely<br>3 = Sometimes<br>4 = Often<br>5 = Very often	Q 3M
**How likely do you see yourself using the LUMOback in the next 6 months?**	1 = Very unlikely<br>2 = Unlikely<br>3 = Not sure<br>4 = Likely<br>5 = Very likely	Q 3M, 12M
**Would you recommend the LUMOback to a friend?**	1 = Yes<br>2 = No	Q 3M
**Did you have any adverse experiences from using the LUMOback?**	1 = Yes<br>2 = No	Q 3M, 12M
**Did you have any adverse experiences from using the LUMOback?**	Text	Q 3M, 12M

**Are there any other comments you would like to make about the LUMOback and/or the LUMOback app?**	Text	Q 3M, 12M

Q = questionnaire; BL = baseline; 3M = 3-month assessment; 12M = 12-month assessment

Workplace strategy use	Response options	Source
**In the past month I have:**<br>• **stood up during a meeting**<br>• **stood up when talking on the phone**<br>• **walked to talk to a colleague rather than sending them an email or phoning**<br>• **walked to the printer that is further away from my office**<br>• **walked to the bathroom that is further away from my office**<br>• **walked to a centrally located bin instead of using my own bin**<br>• **attended a meeting where the chairperson indicated that it was acceptable to stand**<br>• **noticed signage around the office to encourage standing up, sitting less and moving more**<br>• **used prompts at my desk (such as postcards and stickers) to remind me to stand up regularly**<br>• **used computer break software or computer prompts to remind me to stand up regularly**<br>• **eaten my lunch away from my desk**<br>• **used the stairs instead of taking the lift**<br>• **cycled to work**<br>• **walked to work**<br>• **gone for a walk during my lunch break**<br>• **had a walking meeting**<br>• **walked laps of the office to increase my movement**<br>• **participated in group physical activity sessions during work hours**	1 = Never<br>2 = Rarely<br>3 = Sometimes<br>4 = Often<br>5 = Very often	Q BL, 3M, 12M

Q = questionnaire; BL = baseline; 3M = 3-month assessment; 12M = 12-month assessment

Technology questions	Response options	Source
**Q1 What technology do you own or have daily access to?**	Multiple choice of:<br>• Smartphone<br>• iPad or tablet<br>• Laptop or Personal Computer<br>• None of these	Q BL, 3M
**Q2 How confident are you with a smartphone?**<br>***Only asked this question if the following conditions are met:***<br>***Answer was at question 1 'smartphone'***	1 = Not at all confident<br>2 = Slightly confident<br>3 = Confident<br>4 = Very confident<br>5 = Extremely confident	Q BL, 3M
**Q3 How confident are you with an iPad or tablet?**<br>***Only asked this question if the following conditions are met:***<br>***Answer was at question 1 'iPad or tablet'***	1 = Not at all confident<br>2 = Slightly confident<br>3 = Confident<br>4 = Very confident<br>5 = Extremely confident	Q BL, 3M
**Q4 How confident are you with a laptop or personal computer?**<br>***Only asked this question if the following conditions are met:***<br>***Answer was at question 1 'laptop or personal computer'***	1 = Not at all confident<br>2 = Slightly confident<br>3 = Confident<br>4 = Very confident<br>5 = Extremely confident	Q BL, 3M
**Q5 Have you used any other apps or wearable devices to help you be more active?**	1 = Yes<br>2 = No	Q BL, 3M, 12M
**Q6 Please list all the apps and devices you have used to be more active:**	text	Q BL, 3M, 12M

Q = questionnaire; BL = baseline; 3M = 3-month assessment; 12M = 12-month assessment

Musculoskeletal items	Response options	Source
**Q1 Have you at any time during the LAST 1 MONTH had trouble (such as ache, pain, discomfort, numbness) in [body part]?**<br><br>**(if yes asked Qs 2-4)**	1 = No<br><br>2 = Yes	Q BL, 3M, 12M
**Q2 How many days have you had trouble in your [body part] during the last 1 month?**	1 = 0 days<br><br>2 = 1-7 days<br><br>3 = 8-30 days<br><br>4 = everyday	Q BL, 3M, 12M
**Q3 On average, how intense was your pain in your [body part] during the last 1 month on a 0-9 scale? (where 0 means no complaints and 9 means pain as bad as it can be)**	0-9 scale	Q BL, 3M, 12M
**Q4 Have you during the last 1 month been prevented from carrying out normal activities (e.g. job, housework, hobbies) because of trouble from your [body part]?**	1 = No<br><br>2 = Yes	Q BL, 3M, 12M
Asked for each body part including:<br><br>**Neck, shoulders, elbows, wrists/hands, upper back, lower back (small of back), one or both hips/thighs/buttocks, one or both knees, one or both ankles/feet**		
**For shoulders, elbows and wrists/hands**<br><br>**Q1 is instead:**	1 = No<br><br>2 = Yes, but only in the right side<br><br>3 = Yes, but only in the left side<br><br>4 = Yes, in both sides	
