# Supplementary material for: Organizational-Level Strategies With or Without an Activity Tracker to Reduce Office Workers’ Sitting Time: Rationale and Study Design of a Pilot Cluster-Randomized Trial
Source: JMIR Res Protoc. 2016 May 25;5(2):e73. doi: 10.2196/resprot.5438 (PMC4909392; doi:10.2196/resprot.5438)
Supplement: Multimedia Appendix 2 [file resprot_v5i2e73_app2.pdf]

Multimedia Appendix 2: telephone interview questions

These questions will be used in the telephone interviews. Numbers indicate the main questions, with sub-questions indicated with arrows underneath.

1. So, how have you found the study in general?

The following questions are for Group 2 LUMOback participants only.

2. What are your thoughts on the LUMOback in general?

3. Just to confirm you wore the LUMOback during the study period?

4. What were your reasons for wearing or not wearing the LUMOback?

If worn:

5. What did you like about the LUMOback?

6. Was the LUMOback acceptable to wear?

7. What was your initial motivator for using the LUMOback? i.e. improve posture, reduce sitting, because you thought you had to, peer pressure?

8. How did you find the sitting notification prompt?

9. What were some of the barriers to wearing the LUMOback?
   - ➢ At work
   - ➢ At home

10. Did any of your colleagues or team members or your team manager wear a LUMOback?

11. Did this influence your wear/ non-wear of the LUMOback?

12. Do you think your wear/non-wear of the LUMOback influenced your team members? *(asked of team managers only)*

13. Would you wear a different wearable device that monitored your activity and your sitting?

If yes:

- ➢ What sort of qualities would you want in a wearable device? (size, different body placement etc.)

-- the next questions are for all participants (except team managers – questions for managers begin again at Q24) –

Organisational support questions for team members (not team managers)

14. What in particular did your organisation or team manager do to support you standing and moving more at work? (if they did)

15. What could your organisation or team manager have done to be more supportive?

16. Have the organisational norms and/or culture around sitting/standing and moving changed within your work group since participating in the study?

17. What do you find positive/negative about this change?

18. What were the key elements of the intervention that helped you change?

19. What sitting, standing or stepping strategies are you likely to continue using?

20. What could be changed or improved with the intervention?

21. Did you find the overall intervention acceptable?

22. Could you see your workplace taking on any other changes in the short or long term?

23. Did you have any other comments you would like to make about the study or in general?

-- end of interview --

Organisational support questions for team managers only

24. During the study period did you do anything with your team to initiate sitting less, standing or moving more?

If yes:

- what worked/ what didn't?

25. Was there anyone else who initiated change in your team?

If yes:

- what did they do?

26. As a result of the study, have the organisational norms and/or culture around sitting/standing and moving changed within your work group?

27. What do you see as advantages to less sitting at work? Any negatives about this change?

28. What is it going to take for these changes to become sustainable in your group in the long-term?

29. What proportion of your staff do you think would benefit from additional support (emails, or devices) to reduce their sitting time?

30. What were the key elements of the intervention that helped you change?

31. What sitting, standing or stepping strategies are you likely to continue using?

32. Have your use of these strategies influenced others in your team?

33. What could be changed or improved with the intervention?

34. Did you find the overall intervention acceptable?

35. Did you have any other comments you would like to make about the study or in general?

-- end of interview --
