# Supplementary material for: Organizational-Level Strategies With or Without an Activity Tracker to Reduce Office Workers’ Sitting Time: Rationale and Study Design of a Pilot Cluster-Randomized Trial
Source: JMIR Res Protoc. 2016 May 25;5(2):e73. doi: 10.2196/resprot.5438 (PMC4909392; doi:10.2196/resprot.5438)
Supplement: Multimedia Appendix 3 [file resprot_v5i2e73_app3.pdf]

Multimedia Appendix 3: focus group questions

These questions will be used as a guide for the focus groups. It is likely that not all questions will be asked. Numbers indicate the main questions, with sub-questions indicated with arrows underneath.

1. So firstly what things have changed in your workplace since the start of the study to promote standing up, sitting less and moving more?
   - ➢ What caused these changes?

2. Do you now feel more supported by Lend Lease to stand up, sit less and move more?
   - ➢ At which levels of management is it important to have support or endorsement for the program?
   - ➢ Would you be able to name names of any individuals who were particularly important to have on board?

3. Do you now feel more comfortable among your peers to stand up, sit less and move more?

4. Have any changes to the physical environment of Lend Lease occurred since the start of the study?
   - ➢ Have changes to the physical environment affected how much you stand up, sit less and move more?
   - ➢ Were these physical environment changes related to the study?

5. Has your activity and sitting behaviour changed since the start of the study?

➔ If yes:
   - ➢ What caused this change?
   - ➢ Have you any other strategies such as goal setting, reminders, or computer software prompts?
   - ➢ What is it going to take to maintain the use of these individual strategies?

➔ If no:
   - ➢ Have you experienced any other changes?
     Probe: for example changes in your awareness of your sitting or activity levels?
   - ➢ What is it going to take for you to make changes in your behaviour?

6. What is it going to take for these changes to become sustainable in the long term?

7. What further changes do you want to see to support you as an individual?

8. Have you ever worn a wearable device or activity tracker?

- ➔ If yes:
  - ➢ Which ones have you tried?
  - ➢ Why did you start using? [if not the LUMOback]
- ➢ Are you currently wearing an activity tracker?

  If yes:
    - ➢ How long have you worn the tracker for?
    - ➢ How have you found wearing the tracker for this length of time?
    - ➢ What are the reasons for your continued use?

  If no:
    - ➢ How long did you wear the tracker for?
    - ➢ What are the reasons for your discontinued use?
    - ➢ What would help you to wear a tracker for a longer period of time?
    - ➢ Would you wear an activity tracker again?
  - ➢ What would influence you to start wearing again?

- ➔ If no:
  - ➢ Would you like to wear an activity tracker/wearable device?
  - ➢ What things would be important to get you to start wearing?

9. Have you noticed other staff members wear activity trackers or wearable devices?
  - ➢ Does seeing others wear make you want to wear an activity tracker?
  - ➢ Do you think activity trackers promote a culture of health or do you think it's something different? (for example prestige or novelty)

10. There are two ideas on how to use activity trackers, some believe that you need to wear them every day to encourage behaviour change, others believe that you can wear them for a bit and this is enough to encourage behaviour change, what would you prefer?
- ➢ How would each of these approaches change your behaviour?

11. What is needed to promote the ongoing use of activity trackers in general?
